# Evaluating Exposure to VOCs and Naphthalene for Firefighters Wearing Different PPE Configurations through Measures in Air, Exhaled Breath, and Urine

**DOI:** 10.3390/ijerph20126057

**Published:** 2023-06-06

**Authors:** Alexander C. Mayer, Kenneth W. Fent, Andrea F. Wilkinson, I-Chen Chen, Miriam R. Siegel, Christine Toennis, Deborah Sammons, Juliana Meadows, Richard M. Kesler, Steve Kerber, Denise L. Smith, Farzaneh Masoud, Deepak Bhandari, Yuesong Wang, Benjamin C. Blount, Antonia M. Calafat, Gavin P. Horn

**Affiliations:** 1 Division of Field Studies and Engineering, National Institute for Occupational Safety and Health (NIOSH), Centers for Disease Control and Prevention (CDC), Cincinnati, OH 45226, USA; 2 Health Effects Laboratory Division, National Institute for Occupational Safety and Health (NIOSH), Centers for Disease Control and Prevention (CDC), Cincinnati, OH 45226, USA; 3 Division of Science Integration, National Institute for Occupational Safety and Health (NIOSH), Centers for Disease Control and Prevention (CDC), Cincinnati, OH 45226, USA; 4 Fire Safety Research Institute, UL Research Institutes, Columbia, MD 21045, USA; 5 Health and Human Physiological Sciences, Skidmore College, Saratoga Springs, NY 12866, USA; 6 Illinois Fire Service Institute, University of Illinois at Urbana-Champaign, Urbana, IL 61801, USA; 7 Division of Laboratory Sciences, National Center for Environmental Health, Centers for Disease Control and Prevention (CDC), Atlanta, GA 30341, USA

**Keywords:** personal protective equipment (PPE), firefighters, volatile organic compounds (VOCs), breath, urine, benzene

## Abstract

Firefighters are at an increased risk of cancer due to their occupational exposure to combustion byproducts, especially when those compounds penetrate the firefighter personal protective equipment (PPE) ensemble. This has led to questions about the impact of base layers (i.e., shorts vs. pants) under PPE ensembles. This study asked 23 firefighters to perform firefighting activities while wearing one of three different PPE ensembles with varying degrees of protection. Additionally, half of the firefighters unzipped their jackets after the scenario while the other half kept their jackets zipped for five additional minutes. Several volatile organic compound (VOC) and naphthalene air concentrations outside and inside of hoods, turnout jackets, and turnout pants were evaluated; biological (urinary and exhaled breath) samples were also collected. VOCs and naphthalene penetrated the three sampling areas (hoods, jackets, pants). Significant (*p*-value < 0.05) increases from pre- to post-fire for some metabolites of VOCs (e.g., benzene, toluene) and naphthalene were found. Firefighters wearing shorts and short sleeves absorbed higher amounts of certain compounds (*p*-value < 0.05), and the PPE designed with enhanced interface control features appeared to provide more protection from some compounds. These results suggest that firefighters can dermally absorb VOCs and naphthalene that penetrate the PPE ensemble.

## 1. Introduction

Over the past few decades, several studies have demonstrated through air samples that structural fires produce hazardous volatile organic compounds (VOCs) including benzene, toluene, acrolein, styrene, and polycyclic aromatic hydrocarbons (PAHs) like naphthalene [1,2,3], some of which are known (benzene), probable (acrolein, styrene), or possible (naphthalene) carcinogens according to the International Agency for Research on Cancer (IARC) [4]. Several studies have documented firefighter exposures to these hazardous chemicals through exhaled breath or urinary metabolites detected in samples collected after fire responses [5,6,7,8,9,10]. The inhalation route is of concern, especially when firefighters complete job tasks without donning of self-contained breathing apparatus (SCBA) (e.g., exterior operations) or remove their SCBA when compounds are still in the air (e.g., overhaul operations). However, several recent studies [7,8,11] have reported that firefighters have elevated benzene concentrations in breath and urine samples taken post-fire even though the firefighters wore SCBA throughout the fire response, suggesting that dermal absorption contributed to these exposures.

Some studies have found that firefighters have an increased risk for certain types of cancer [12,13,14]. Daniels et al. (2015) found a relationship between the number of fire runs and leukemia and fire hours spent on-scene and lung cancer [15], strengthening the link between occupational exposures and cancer outcomes among firefighters. In 2022, IARC classified firefighting as a known carcinogen to humans (Group 1) based on sufficient evidence of increased risk of bladder cancer and mesothelioma [16].

Historically, firefighting personal protective equipment (PPE) was designed with the primary function of providing protection from thermal hazards while also providing protection against abrasion and body fluids. In recent years, as the exposure risks have been identified, PPE manufacturers have developed new designs for the firefighting PPE ensemble (e.g., particulate-blocking hoods, PPE with physical barriers such as additional overlapping material at the ensemble interface locations) aimed at reducing exposure to combustion byproducts. Some recent studies have shown that, while combustion byproducts can still permeate or penetrate the traditional firefighter turnout jacket design, the current bunker gear is moderately effective at reducing exposures to chemicals like PAHs—many of which will exist primarily as particulate [17]. Additionally, Wingfors et al. (2018) reported that total PAH air concentrations were 146 times lower when measured inside the base layers (i.e., clothing worn by firefighters under turnout jackets, typically a short or long sleeve t-shirt and shorts or pants) compared to outside the jackets [18]. However, the protection provided by base layers may depend on the properties of the chemical. Compared to the total PAHs, naphthalene was found to have a lower protection factor (mean protection factor of 49) in the same study. Porous base layers will likely have a smaller effect on vapors than particulate. The protection provided by PPE suggests that base layers may provide additional protection from the ingress of contaminants contacting skin, but the protection largely depends on the volatility of the compound in question.

While the relative reduction in PAH exposure is encouraging, more recent studies have found that more volatile chemicals like benzene penetrate through and/or around turnout jackets worn by firefighters, often reaching levels close to those reported outside the jackets [11]. It is reasonable to expect volatile chemicals like benzene (and lower molecular weight PAHs such as naphthalene) to penetrate around turnout pants as well as around and through protective hoods. Indeed, a recent study measured PAHs on wipes taken from the neck region of firefighters wearing particulate-blocking or traditional knit hoods after simulating fireground operations in a fireground exposure simulator (FES). While particulate-blocking hoods offered more protection from PAHs than traditional knit hoods, ingress still occurred [19]. This is not entirely unexpected, as particulate-blocking hoods were not designed to be vapor tight [20,21] and the hood-facepiece and hood-jacket interfaces may still provide a pathway for contamination to reach the neck skin.

The permeation or penetration of chemicals like benzene through the firefighter PPE ensemble increases the likelihood of dermal absorption. Mayer et al. (2022) found that benzene exhaled breath concentrations increased significantly post-fire compared to pre-fire for firefighters wearing SCBA and PPE ensembles that included traditional knit hoods or newer particulate-blocking hoods [11]. VOCs like benzene and low molecular weight PAHs like naphthalene typically remain in the vapor phase, potentially allowing them to be absorbed through the skin. It has been shown that small amounts (<1%) of benzene may be absorbed directly through skin [22,23]. Therefore, it’s possible firefighters may dermally absorb a portion of these chemicals if they permeate or penetrate the PPE ensemble and recondense on the skin and/or PPE. This has led to questions about whether quickly unzipping turnout gear post-fire can reduce dermal exposure to contaminants trapped in the PPE ensemble.

Some have hypothesized that wearing base layer shorts instead of base layer long pants under turnout pants may reduce heat stress for firefighters responding to fires in a warm environment, as is common in certain parts of the United States [24]. Additionally, it has been postulated that unzipping turnout gear quickly post-fire may reduce potential dermal exposure to chemicals trapped inside the gear. However, to our knowledge, no studies have examined either of these questions. This study sought to investigate the effects of these PPE-related practices on exposures to VOCs and naphthalene through two objectives. First, we evaluated exposure associated with different PPE base layers (short sleeve and shorts; long sleeve and pants) worn under traditional NFPA 1971-compliant turnout gear and an experimental PPE ensemble design containing a one-piece moisture barrier layer (eliminating jacket-pant interface and introducing tighter jacket-hood, jacket-glove and pant-boot interfaces) with firefighters undergoing training exercises in smoke and elevated ambient temperatures. Second, we evaluated whether unzipping turnout jackets immediately after exiting a live-fire training structure affected firefighters’ exposure. The chemicals quantified by each PPE configuration and zip status included VOCs (i.e., benzene, toluene, ethylbenzene, xylenes, and styrene) and naphthalene outside and inside hoods, turnout jackets, and turnout pants worn by firefighters; biologically-absorbed chemical measurements in breath and urine samples taken before and after the fireground operations; and comparisons between external (airborne) and internal (biological) exposures.

## 2. Materials and Methods

### 2.1. Study Population and Design

This study was performed at the University of Illinois Fire Service Institute (IFSI) with collaboration from the National Institute for Occupational Safety and Health (NIOSH) and Firefighter Safety Research Institute (FSRI) of UL Research Institutes. Institutional Review Board (IRB) approval was obtained from the University of Illinois at Urbana-Champaign and CDC/NIOSH.

Participants were recruited through a local and statewide network of firefighters. Twenty-three firefighters, 21 men and two women, participated in three standard training scenarios. Participants ranged in age from 18–42 and the participants’ median body mass index (BMI) was 30.2 (range = 20.3–39.6). Firefighters were cleared by their department, fit-tested for SCBA, and took a pre-screening questionnaire. Firefighters with known underlying cardiac or gastrointestinal conditions, who were pregnant, or used tobacco were excluded from participating in the study.

Using a repeated measures design, firefighters completed three separate but identical training scenarios in a FES that permitted standard exposure over a period of three days, wearing a different PPE configuration on each day. Twenty-two of the 23 firefighters participated in all three scenarios, while one firefighter participated in two scenarios. Up to six firefighters (three firefighters per chamber) per scenario performed simulated fireground operations in the FES. The fuel package and simulated firefighting activities for all scenarios were uniform and conducted utilizing the FES. All PPE was brand-new at the beginning of the study.

**Figure 1:**
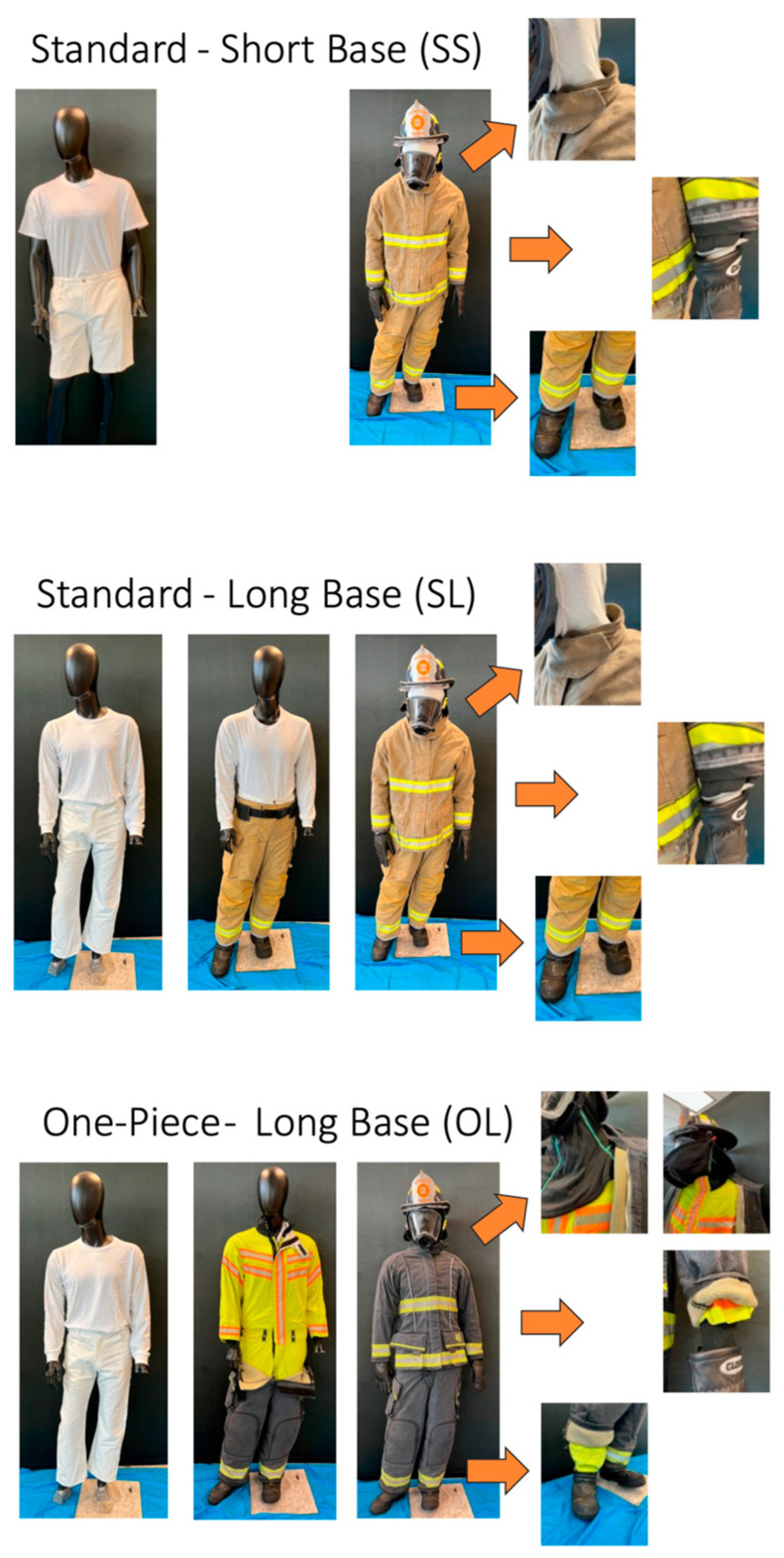
The three PPE configurations worn by firefighters including: (SS) standard, commercially available PPE with knit hood and cotton short-sleeve shirt and shorts base layer, (SL) standard, commercially available PPE with knit hood cotton long-sleeve shirt and long pants base layer, and (OL) interface control PPE (PPE with enhanced features designed to reduce transmission of contaminants through jacket-pant, jacket-particulate-blocking hood, jacket-glove and pant-boot interfaces) with cotton long-sleeve shirt and long pants base layer (OL).

Firefighters were provided three PPE and base layer configurations, which were fitted based on their chest and waist sizes. The three configurations included:

1. Standard, commercially available NFPA 1971-compliant PPE with cotton short-sleeve shirt and shorts base layer (SS) and knit hood,
2. Standard, commercially available NFPA 1971-compliant PPE with cotton long-sleeve shirt and long pants base layer and knit hood (SL), and
3. Interface control PPE (experimental PPE ensemble designed with enhanced interface control features between jacket and pants (one-piece moisture barrier layer) and tightened interface between particulate-blocking hood and jacket, jacket and gloves, and boots and pants)) with cotton long-sleeve shirt and long pants base layer and particulate-blocking hood (OL; Figure 1).

The SS configuration and SL configuration were compared to evaluate differences in exposure based on base layer (short sleeve shirts and shorts vs. long sleeve shirts and pants), and the SS and SL configurations were compared against the OL configuration to characterize differences in the interface PPE design (standard PPE vs. the interface control PPE). Comparisons are made between the three configurations in the subsequent analyses.

The FES has been described in detail previously [11,25]. Briefly, the FES is built from a shipping container measuring 2.4 m wide, 2.9 m tall, and 12.2 m long. The container is divided into three sections where the middle section (3.1 m long) serves as a controlled combustion chamber and the chambers on the left and right side are equipped to train six firefighters simultaneously in a low visibility smoke environment. Smoke was generated from burning a popular residential sofa in the combustion chamber; the smoke was ducted into the training chambers with firefighters on each side.

To begin each training scenario, a road flare was ignited and placed in the center of the sofa, loading doors were immediately closed, resulting in the only existing leakage paths in the combustion chamber for ventilation. After ignition, the fire was allowed to ventilate for 6 min prior to fire suppression from a water hose. Timing of activities and sofa ignition were coordinated to mimic conditions of realistic firefighting operations (Table 1). An experienced researcher with firefighting experience was present in each side of the smoke chamber to serve as a safety escort throughout the training scenarios.

Following each scenario, half of the firefighters quickly unzipped their jacket immediately after exiting the structure. All firefighters were then transported to the data collection area and the other half of the firefighters kept their jacket zipped (for approximately 5 min) until they doffed their PPE at the data collection area (unzip vs. zip).

### 2.2. Personal Air Sampling

Personal air sampling was similar to our previous study [11]. Briefly, air sampling pumps and media (6 × 70-mm glass charcoal tubes) were attached to firefighter’s SCBA at chest height to assess VOCs and naphthalene concentrations outside the gear (outside samples). Additionally, active air samples were taken underneath firefighter PPE (but above base layer) in the jacket pocket for PPE configurations SS and SL and attached to the suspenders at chest height for PPE configuration OL (inside jacket samples). Pre-sampling calibration flow rates were based on the target flow rates of 0.1 L/min for charcoal tubes and all pumps had post-sampling flow rates that were within 5% of the pre-sampling calibration flow rate. Field blanks were collected from each scenario. Following each scenario, media were collected and placed in a refrigerator where they were kept until analysis. To assess VOCs (benzene, toluene, ethylbenzene, xylenes, and styrene) and naphthalene, charcoal tubes were analyzed using NIOSH Method 1501. The sampling time for outside personal air samples was 11 min and the air sampling time for inside jacket personal air samples was 16 min.

To assess the magnitude of VOCs and naphthalene inside protective hoods and turnout pants, thermal desorption (TD) passive air samplers (Tenax TA thermal desorption tubes) were attached inside the PPE, but above firefighters’ base layers on the neck (inside hood samples) and on pants/shorts (inside pants samples). For each scenario, samplers were attached inside the PPE for one SL configuration, one SS configuration, and two OL configurations. TD passive field blanks were also collected for each scenario. Following each scenario, as firefighters doffed their PPE, TD samplers were capped as quickly as possible and stored in a refrigerator until analysis. Samples were desorbed and analyzed for VOCs and naphthalene following EPA TO-17 [26]. Passive sampling time for inside gear samples was 16 min. Diffusion rates used in this study (1.3 ng/ppm*min for benzene, 1.67 ng/ppm*min for toluene, 2.4 ng/ppm*min for styrene) were reported in ISO Standard 16017–2 and diffusion rates used for naphthalene (2.14 ng/ppm*min) were reported in Lindahl et al. (2011) [27]. Ethylbenzene and xylenes outside and inside gear samples had detection rates below 50% so results were not reported in this manuscript. Active and passive samples collected from three firefighters wearing the OL configuration (three inside hood, three inside jacket, three inside pants) were removed from analyses because researchers inadvertently placed sampling media in an incorrect location.

### 2.3. Exhaled Breath Sampling

Breath samples were collected using the BioVOC™ (Markes International, Inc., Cincinnati, OH, USA), from firefighters before and immediately after doffing gear following each fire scenario (*n* = 128). Eight samples were removed from analyses (six because of the incorrect sampling location on firefighters wearing the OL configuration and two because of lab error). Additionally, one firefighter provided breath samples before and after only two scenarios. Both pre and post samples were collected outside in a tent. Post-exposure breath samples were collected as soon as operationally feasible following doffing of gear and SCBA. Firefighters were instructed to inhale deeply and then forcefully exhale their breath into the BioVOC™ twice which resulted in 258 mL of breath per sample. The breath sample was pushed into Markes TD tubes (Carbograph 2TD/1TD dual bed tubes) and stored in a refrigerator. One field blank was collected for each collection period. Breath samples were analyzed for VOCs (benzene, toluene, ethylbenzene, xylenes, and styrene) [28]. Ethylbenzene, xylenes, and styrene exhaled breath samples had detection rates below 50% so results were not reported in this manuscript.

### 2.4. Urine Sampling

Firefighters provided spot urine samples pre-firefighting (*n* = 68), 3-h post scenario (*n* = 68), and 6-h post scenario (*n* = 35). Firefighters were encouraged to hydrate throughout the scenarios, including after the scenario. Participants were provided sterile 120-mL specimen collection cups, instructed to wash their hands, and provide a clean-catch sample. Samples were aliquoted into tubes and stored at −70 °C pending PAH metabolite quantification and at −80 °C for those pending cotinine and creatinine measurements. Urine specific gravity was measured immediately following sample collection using a hand-held refractometer (Atago, Uricon-Ne, Mumbai, India). VOC metabolite analysis was performed using ultrahigh performance liquid chromatography coupled with electrospray ionization tandem mass spectrometry (UPLC-ESI-MS/MS) with some modification to the specific analytes of interest outlined in a published procedure [29]. Benzene biomarkers, phenylmercapturic acid (PhMA) and muconic acid (MUCA) were measured by using a separate UPLC-ESI-MS/MS method [30] (Table 2). Creatinine was measured using a Vitros Autoanalyzer (Johnson & Johnson, New Brunswick, NJ, USA) with a Vitros CREA slide. Cotinine was measured to quantify potential tobacco usage and exposure to environmental tobacco smoke which can be a source of VOC exposure [31], using the Immulite^®^ 2000 analytical platform (Siemens Corporation, Washington, DC, USA). After enzymatic hydrolysis of conjugated PAH metabolites in 100 µL urine, the target metabolites were quantified by online solid phase extraction coupled with high performance liquid chromatography-isotope dilution tandem mass spectrometry [32]. Limits of detection (LODs) were 60 ng/L for 1-hydroxynaphthalene (1-NAP) and 90 ng/L for 2-hydroxynaphthalene (2-NAP).

### 2.5. Data Analysis

**Figure 2:**
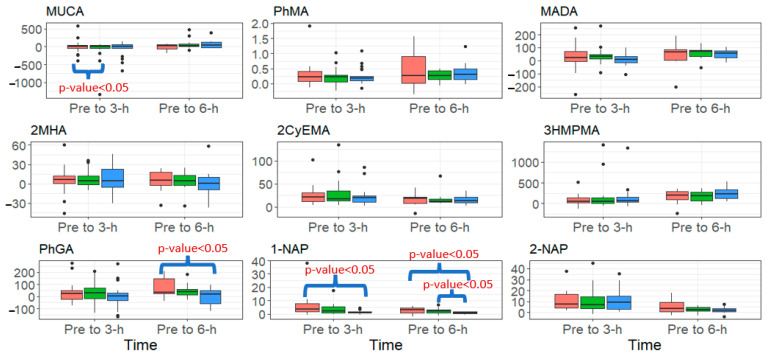
Summary of the change in urinary biomarker concentrations (μg/g creatinine) from pre to 3-h and pre to 6-h post-fire, stratified by PPE configuration (red = SS (Standard- Short Base), green = SL (Standard- Long Base), blue = OL (One-piece Long Base) for analytes that increased significantly over both of those periods. Black dots signify outliers.

Descriptive statistics (number of samples (N), number of sample concentrations below LOD, mean, median, and range) are reported for air concentrations, stratified by firefighting PPE ensemble (configurations), sampling location, and zip status (Table 3). The same descriptive statistics (replacing mean with geometric mean (GM)) are presented for exhaled breath concentrations by configuration and zip status and for VOC and naphthalene urinary metabolite creatinine-adjusted concentrations by collection period. Workplace protection factors (WPFs), which estimate the level of protection for a worker in a hazardous environment, were calculated by dividing the median outside PPE concentration by the median result found inside the respective PPE location. Higher WPFs indicate a higher level of protection. Non-detectable air, exhaled breath, and VOC and naphthalene metabolite concentrations below the LOD were imputed using the β-substitution method [33], which assigned values to the non-detects based on the distribution of the uncensored data. To compare with the VOC and naphthalene metabolite results, we calculated 95th percentile concentrations of VOC and naphthalene metabolites based on a representative sample of the U.S. general population aged 18–55 years from the 2013–2014 and 2015–2016 National Health and Nutrition Examination Survey (NHANES). To visualize the VOC metabolite results, box and whisker plots with minimum, 25th percentile, median, 75th percentile, and maximum were created for the stratifications of PPE configuration and collection period (Figure 2).

A mixed model with individual firefighter as a random effect was utilized to account for statistical correlation among repeated measures from the same firefighter [34]. Univariable analyses were performed using the exhaled breath geometric mean (GM) concentration from pre- to post-fire as the dependent variable and investigating whether the change in exhaled breath GM concentrations from pre- to post-fire was significantly different from zero by analyte, PPE configuration, or zip status. Multiple comparisons were also conducted to determine if the magnitude of concentration differences from pre- to post-fire were different among configurations and zip statuses. A mixed model with log-transformed urinary metabolite concentration as the dependent variable was also used to examine whether the change in urinary GM concentration from pre-fire to 3-h post-fire and from pre-fire to 6-h post-fire was significantly different from zero. Finally, we conducted an association analysis, through a mixed model, to examine outside and inside gear air concentrations and exhaled breath concentrations, and outside and inside gear air concentrations and urinary metabolite concentrations. Statistical tests were two-sided at the 0.05 significance level. All analyses were performed in SAS version 9.4 (SAS Institute, Cary, NC, USA).

## 3. Results

### 3.1. Benzene, Toluene, Styrene, and Naphthalene Personal Air Concentrations Outside and Inside Firefighter PPE

Table 3 summarizes the benzene, toluene, styrene, and naphthalene air concentrations from outside and inside hoods, turnout jackets, and turnout pants. Turnout jackets provided more protection than turnout pants, and both turnout jackets and turnout pants provided more protection from benzene than protective hoods. Benzene WPFs for hoods were consistently around 1, indicating no protection. Benzene permeated or penetrated inside hoods, turnout jackets, and turnout pants, regardless of PPE configuration or zip status. Notably, zip status did appear to impact benzene concentrations inside turnout jackets, as the median WPFs for those who unzipped their turnout jacket were consistently higher than those that kept their turnout jacket zipped for an extra 5 min (SS: 1.61 zip/1.72 unzip; SL: 1.17 zip/2.46 unzip; OL: 1.58 zip/2.51 unzip). The findings for toluene and styrene regarding zip status were similar, though their WPFs were higher by comparison (toluene SS: 1.68 zip/1.95 unzip; SL: 1.26 zip/3.17 unzip, OL: 1.73 zip/3.78 unzip; styrene SS: 32.8 zip/33.1 unzip; SL: 1.65 zip/9.28 unzip; OL: 3.52 zip/35.7 unzip). The experimental PPE design focused on reducing interface leakage was successful as SL was found to have a significantly higher GM concentration of styrene inside turnout jackets than OL (*p*-value = 0.026).

Naphthalene WPFs were higher for the turnout jacket and turnout pants compared to the hoods for the standard PPE (SS and SL), but not for the experimental PPE that included a particulate-blocking hood and measures to control leakage at ensemble interfaces (OL). However, the WPFs were much higher for naphthalene (WPF range for hoods, turnout jackets, and turnout pants are 5.29–302, 10.3–122, and 39–302, respectively), indicating a higher level of protection from this compound. When we compared naphthalene concentrations by PPE configuration, the GM concentration for OL was significantly lower than for SS and SL (*p*-values = 0.003 and < 0.001, respectively). This appeared to be largely driven by the detection frequency for inside gear samples, as the majority of the stratified sample results for the OL condition had a detection frequency under 25%. In fact, naphthalene was only detected in 5 out of 54 samples collected while firefighters were wearing the OL PPE, compared to 33 out of 49 when firefighters were wearing the SL PPE configuration.

### 3.2. VOC Exhaled Breath Concentrations and Their Association with Personal Air Concentrations

Table 4 outlines the pre- and post-fire benzene and toluene exhaled breath concentrations, stratified by PPE base layer and design configuration and zip status. Benzene and toluene exhaled breath concentrations increased significantly from pre- to post-fire for all PPE configurations and zip statuses. Firefighters wearing the OL configuration appeared to have a moderately lower difference of pre- and post-fire benzene exhaled breath concentrations compared to the other two PPE configuration, but the results were not statistically significant. Zip status did not significantly impact the difference of pre- and post-fire benzene or toluene exhaled breath concentrations. Regression modeling results (for all PPE configuration combined) showed positive associations between outside gear benzene air concentrations and exhaled breath benzene concentrations, and between inside turnout jacket benzene air concentrations and exhaled breath benzene concentrations (both *p*-values < 0.001) (Table 5). The change in pre- to -post-fire exhaled breath benzene concentrations was associated with both inside turnout jacket samples and outside hood samples, but the association was higher with inside turnout jacket samples (47% higher). After stratifying by PPE configuration, these regressions remained statistically significant for SS and OL configurations.

### 3.3. VOC and Naphthalene Urinary Metabolite Concentrations and Their Association with Personal Air Concentrations

Table 6 summarizes the VOC and naphthalene urinary metabolite results, stratified by collection period. Several urinary metabolites (corrected for creatinine) increased significantly from pre-fire to 3- and 6-h post-fire, including benzene derivatives trans,trans-muconic acid (MUCA) and N-acetyl-S-(phenyl)-L-cysteine (PhMA), styrene/ethylbenzene derivatives mandelic acid (MADA) and phenylglyoxylic acid (PhGA), xylene derivative 2-methylhippuric acid (2MHA), acrylonitrile derivative N-acetyl-S-(2-cyanoethyl)-L-cysteine (2CyEMA), crotonaldehyde derivative N-acetyl-S-(3-hydroxypropyl-1-methyl)-L-cysteine (3HMPMA), and naphthalene derivatives 1-hydroxynaphthalene (1-NAP) and 2-hydroxynaphthalene (2-NAP). Most (over 50%) metabolites’ 6-h GM concentrations were higher than both the 3-h and pre-fire concentrations. Overall, both pre- and post-fire GM concentrations for the majority (over 75%) of metabolites were below the 95% percentile general population concentrations, with the exception of 2CYeMA. Notably, MUCA pre- and post-fire results were above the American Conference of Governmental Industrial Hygienists (ACGIH) biological exposure index (BEI) (500 µg/g creatinine) on several occasions, and this was true for firefighters wearing all three PPE configurations. However, PhMA concentrations, a more specific biomarker of benzene exposure, were well below the applicable BEI for pre- and post-fire concentrations (25 µg/g creatinine) [35].

Figure 2 presents the change in urinary metabolites from pre- to 3-h and 6-h post-fire for metabolites that increased significantly over this period, stratified by PPE configuration. Firefighters wearing the SS configuration had a significantly higher change from pre- to 6-h post-fire for PhGA and 1-NAP compared to firefighters wearing the OL configuration (*p*-value < 0.05), but no significant differences were found between SS and SL. Additionally, firefighters wearing the SS configuration had a significantly higher change from pre- to 3-h post-fire for 1-NAP compared to the OL configuration (*p*-value < 0.05). Lastly, firefighters wearing the SS configuration had a significantly higher change from pre- to 3-h post-fire for MUCA compared to SL (*p*-value < 0.05). We found no differences in urinary metabolites by zip status.

Regression analyses results (for all PPE configurations combined) demonstrated positive associations between outside turnout gear benzene air concentrations and PhMA concentration differences from pre-fire to 3-h post-fire, and between inside turnout jackets benzene air concentrations and PhMA concentration differences from pre- to 3-h post-fire (both *p*-values < 0.001) (Table 5). After stratifying for PPE configuration, these regressions remained statistically significant (*p*-value < 0.05) for all but the outside SL gear benzene concentrations and their relationship to the change in PhMA urinary concentrations.

## 4. Discussion

This study evaluated how well three turnout gear PPE and base layer configurations protected firefighters from the ingress of VOCs and naphthalene. Additionally, this study evaluated the effectiveness of quickly unzipping the firefighter turnout jacket post-fire to reduce the exposure to VOCs and naphthalene that may be trapped inside the PPE ensemble after a fire response. Our results suggest that VOCs (benzene, toluene, styrene) and naphthalene can permeate or penetrate all three PPE configurations, and that firefighters absorb these compounds that penetrate the PPE ensemble.

### 4.1. Comparing Personal Air Concentrations Taken Outside the Gear and Inside the Hood, Jacket and Pants, Stratified by PPE Configuration and Zip Status

Overall, benzene air concentrations from this study (medians 13,432–76,923 ppb) were lower but within the same order of magnitude as benzene concentrations from our previous study that had firefighters conducting similar scenarios in the same FES with the same fuel load (medians 62,200–75,900 ppb) [11]. Nearly all outside and inside samples (except two samples taken inside firefighter pants) were above the NIOSH short term exposure limit (STEL) for benzene of 1000 ppb [36], including samples taken in the jacket or breathing zone region. Toluene and styrene air concentrations were similar to previous publications [1,11] and measurements were below applicable NIOSH STELs (styrene STEL = 100,000 ppb; toluene STEL = 150,000 ppb) [36].

When we stratified by sample location, the data suggest that hoods provide less protection from benzene, toluene, styrene, and naphthalene compared to pants, followed by jackets. Benzene WPFs calculated from samples taken inside the hood were below 1, indicating no protection from benzene. This is not altogether surprising given that the hood is one of the weaker aspects of the PPE ensemble from an exposure reduction perspective [37]. This is an exposure pathway that could be more fully evaluated, especially because chemicals may more readily absorb through the thinner neck skin compared to other parts of the body where skin is thicker [38,39].

Although jackets and, to a lesser extent pants, appeared to protect the firefighter from VOCs and naphthalene better than hoods, ingress still occurred. However, quickly unzipping the jacket post-fire did appear to moderately reduce benzene air concentrations. By contrast, firefighters who kept their jacket zipped until doffing their PPE ensemble (about a five-minute difference) saw protection factors that were closer to results reported in our previous study for benzene [11]. Styrene, toluene, and naphthalene all saw similar increases in WPFs for firefighters that unzipped their jacket. Surprisingly, unzipping the jacket appeared to even reduce VOC and naphthalene concentrations inside hoods–possibly by allowing the bottom of the hood to be less tight against the neck and shoulders. Overall, it appears that quickly unzipping the firefighter turnout jacket may be an easy and effective way to reduce the concentration of trapped contaminants that can be dermally absorbed. This is particularly important for benzene which we measured at levels inside gear close to what was found outside the gear. However, this finding could be seen as a contradiction to the current preliminary exposure reduction (PER) practice of remaining on SCBA and fully enclosed in PPE until PER (decontamination) is completed post-fire. Future research could explore the impact of having firefighters unzip and air out their jackets and then possibly rezip their jackets prior to PER.

When we compared the PPE and base layer configurations, we expected to see lower VOC concentrations inside the gear for firefighters wearing the OL configuration compared to the other two configurations (SS and SL). Overall, the results were mixed and no significant differences were found between the PPE configurations for benzene and toluene. However, WPFs for styrene were consistently higher for the OL configuration compared to the SL configuration. The difference between PPE configurations was even more stark for naphthalene, as the WPF for the OL configuration was significantly higher than both SS and SL configuration. These results are consistent with our previous work which found the PPE ensemble to be better at protecting from naphthalene compared to benzene and toluene [11]. The particulate-blocking hoods used in the OL configuration had no protective effect against benzene or toluene, but did appear to reduce ingress of styrene and naphthalene compared to the knit hoods used in the SS and SL configurations. Overall, our data suggest that PPE protection against the compounds in question is likely to increase with decreasing vapor pressure (benzene > toluene > styrene > naphthalene). It is important to note that while the experimental OL PPE ensemble designed with enhanced interface control features may be moderately effective at reducing exposures, firefighters anecdotally reported trouble quickly donning and also reported being uncomfortably warm and sweating profusely while wearing this ensemble. Heat stress and wearability/functionality are important factors to consider when introducing exposure mitigation strategies like new PPE designs.

### 4.2. Evaluating Exhaled Breath and Urinary Metabolite Concentrations and Their Association with Personal Air Concentrations, Stratified by PPE Ensemble

Benzene exhaled breath concentrations increased significantly from pre- to post-fire, regardless of zip status or PPE configuration. This result is similar to our previous studies [8,11] that found significant increases in benzene exhaled breath concentrations from pre- to post-fire. We also examined the relationship between benzene concentrations measured outside and inside jackets and breath concentrations. Both outside and inside air samples were positively associated with the change in exhaled breath concentrations from pre- to post-fire.

Toluene exhaled breath concentrations also increased significantly from pre- to post-fire. Our previous manuscript [11] found toluene exhaled breath concentrations only significantly increased from pre- to post-fire for firefighters wearing new knit hoods (no significant increase was found for those wearing new and laundered blocking hoods). As such, it appears that either (A) firefighters in the current study absorbed higher concentrations of toluene during the scenarios or (B) researchers in the current study were better at capturing post-fire exhaled breath samples in a timely manner. Indeed, an effort was made to capture post-fire samples as quickly as possible after firefighters doffed their gear (within 5 min) compared to upwards of ten minutes in the previous study. If we compare 3-h post-fire toluene urinary concentrations between the two studies (firefighters from previous study = 7.23 µg/g creatinine vs. 6.01 µg/g creatinine in the current study), it appears that the increase in toluene post-fire exhaled breath concentrations is likely a function of prompt sample collection.

Median values of several other urinary metabolites increased significantly from pre-fire to 3-h and 6-h post-fire, including metabolites of benzene (MUCA and PhMA), styrene/ethylbenzene (MADA and PhGA), xylene (2MHA), acrylonitrile (2CyEMA), crotonaldehyde (3HMPMA), and naphthalene (1-NAP and 2-NAP). This is the first time, to our knowledge, that metabolites of acrylonitrile and crotonaldehyde (both of which are Group 2B chemicals according to IARC) [40,41] have been quantified in firefighters’ urine post-fire. Additionally, several metabolites of toluene (N-acetyl-S-(benzyl)-L-cysteine (BzMA)), xylene (3-Methylhippuric acid + 4-Methylhippuric acid (3MHA + 4MHA)), and 1,3-butadiene (N-acetyl-S-(4-hydroxy-2-buten-1-yl)-L-cysteine (4HBeMA)) increased from pre-fire to 3-h post fire, and metabolites of acrolein (N-acetyl-S-(3-hydroxypropyl)-L-cysteine (3HMPA) and N-acetyl-S-(2-carboxyethyl)-L-cysteine (2CoEMA)) and acrylonitrile (N-acetyl-S-(2-hydroxyethyl)-L-cysteine (2HEMA) increased significantly from pre-fire to 6-h post-fire. Urinary concentrations of naphthalene metabolites are similar to previous publications [8,42] which also reported firefighters’ urinary concentrations of PAH metabolites to increase significantly from pre- to post-fire. Additionally, the VOC results are fairly consistent with our previous manuscript [7] that sampled urine from firefighters, firefighter students, and firefighter instructors responding to live structure and training fires and found MUCA, PhMA, MADA, BzMA, 2MHA, 4HBeMA, and 3MHA + 4MHA increased significantly from pre-fire to 3-h post-fire. In the previous manuscript, we hypothesized that because the elimination half-lives for some of the VOC metabolites are several hours, the 3-h post-fire sampling may not have captured the peak excretion period for some VOCs [7]. Results from the current study appear to support this theory, as several metabolites (e.g., 3HPMA, MUCA, PhMA, MADA, 3HMPMA PhGA) had higher 6-h concentrations compared to 3-h concentrations.

We also compared our results to the NHANES general population concentrations for smokers and non-smokers. The majority of the metabolites were below the 95th percentile for non-smokers, though 2CyEMA (acrylonitrile metabolite) median 3- and 6-h concentrations were above this threshold (3-h median = 23.87 µg/g creatinine; 6-h median = 19.24; general population 95% percentile = 8.69). Comparisons were also made to applicable BEIs, and several MUCA (benzene metabolite) pre and 6-h post-fire samples were above the BEI (500 µg/g creatinine). However, sorbic acid found in preserved food can contribute to MUCA concentrations [43]. This, coupled with the fact that MUCA concentrations were elevated pre-shift and that PhMA (a better biomarker of benzene exposure) concentrations were well below the applicable BEI (25 µg/g creatinine) suggest the high MUCA concentrations reported here are not related to benzene exposure as part of the firefighting scenarios.

When we stratified urinary concentrations by zip status, we found no statistically significant differences in urinary concentrations were found between firefighters who unzipped their jackets quickly after the fire and those who kept their jackets zipped (for approximately 5 more mins) until they doffed their entire gear. Though the results were not significant, MUCA and PhMA 3-h post-fire median concentrations were lower for firefighters who unzipped their jackets compared to those who kept them zipped (MUCA median 3-h post-fire zipped = 85.0 µg/g creatinine/unzipped = 75.1 µg/g creatinine; PhMA median 3-h post-fire zipped = 0.41 µg/g creatinine/unzipped = 0.30 µg/g creatinine).

Lastly, we stratified urinary concentrations by PPE configuration for metabolites that had significant increases from pre- to 3-h and 6-h post-fire (Figure 2). Firefighters wearing SS had a significantly larger change from pre- to 6-h post-fire for some metabolites (e.g., 1-NAP and PhGA) compared to urine samples from firefighters wearing the OL configuration. Firefighters wearing the SS configuration had shorts and short sleeve shirts, which may have increased the potential for contaminants penetrating the gear to contact skin. We compared skin permeation coefficient estimates for styrene, toluene, and benzene (5.5 × 10^−2^, and 4.5 × 10^−2^, 2.1 × 10^−2^ cm/h), which suggest that absorption of these compounds is possible [44]. Naphthalene has been estimated to have a higher absorption rate (6.9 × 10^−2^ cm/h), so it is not surprising that we see larger differences between SS, SL and OL for 1-NAP [44]. Overall, it appears the OL design modification was more impactful on exposure reduction compared to the base layer modification (SS vs. SL). However, wearing short base layers has the potential to increase the absorption of some of the chemicals measured here. Regardless, the differences between the 3 configurations were negligible for several of the metabolites (e.g., benzene, styrene) quantified here.

### 4.3. Limitations

This study has some limitations to consider. Sample sizes for some of our stratifications for air samples were relatively small, so statistical power was somewhat limited in the comparisons. This also limited our ability to analyze potential interactions between PPE configurations and zip status. We also did not analyze air samples for a host of chemicals whose biomarkers were measured in urine (e.g., acrolein, 1,3-butadiene, crotonaldehyde, acrylonitrile). Some of these compounds (e.g., acrolein, 1,3-butadiene) have been observed on the fire scene in the past, and future work could sample for the others (e.g., crotonaldehyde, acrylonitrile) in addition to other common contaminants found on the fireground including synthetic compounds like flame retardants and phthalates. In addition, firefighters in this study showered within 10 min after doffing their gear, per best practices. Showering so quickly is not always possible. Exposures could have differed had the firefighters taken longer to shower and is another important factor to study in the future. Firefighting activities in the FES are designed to simulate typical firefighting work, but do not capture all possible firefighting activities and movements or chemicals and concentrations that may be released in all fire ground responses. Furthermore, while smoke exposure is relatively short, it is a relatively high concentration and ambient high-pressure environment, which will not simulate all firefighting situations, particularly for those working outside of a structure. Future studies could characterize exposures for firefighters responding to emergency fire responses to better understand how these exposures compare in duration and magnitude.

## 5. Conclusions

Benzene, toluene, styrene, and naphthalene all penetrated the three sampling areas (hoods, jackets, pants) for all three PPE configurations (SS, SL, OL). However, quickly unzipping the firefighter turnout jacket appeared to lower chemical concentrations inside the jacket. Additionally, benzene and toluene exhaled breath concentrations increased from pre- to post-fire, regardless of PPE configuration. Several urinary metabolites of VOCs and naphthalene also increased significantly (*p*-value < 0.05) from pre-fire to 3-h and 6-h post-fire, including metabolites of benzene (PhMA median pre-fire = 0.12, 3-h post-fire = 0.33, 6-h = 0.54 µg/g creatinine), styrene/ethylbenzene (MADA median pre-fire = 115.9, 3-h = 136, 6-h 144 µg/g creatinine), xylene (2MHA median pre-fire = 12.2, 3-h post-fire = 21.5, 6-h = 16.1 µg/g creatinine), acrylonitrile (2CyEMA median pre-fire = 3.05, 3-h 23.7, 6-h 19.3 µg/g creatinine), and crotonaldehyde (3HMPMA median pre-fire = 143, 3-h = 245, 6-h 381 µg/g creatinine). This is the first manuscript to report significant increases from pre- to post-fire for urinary metabolites of acrylonitrile and crotonaldehyde. We also found an association between personal air benzene concentrations and the change in exhaled breath concentrations and PhMA urinary concentrations from pre- to post-fire. Overall, results from our study show that benzene, toluene, styrene, and naphthalene penetrate the sampling areas (hoods, turnout jackets, and turnout pants). Given that firefighters for this study breathed through SCBA throughout the entire response, it appears that dermal absorption is an important route of exposure for some of these combustion byproducts. PPE protection against compounds appears to increase with decreasing vapor pressure of the chemicals. The PPE designed with enhanced interface control features (OL) appeared to provide the most protection from the chemicals measured compared to those wearing traditional PPE (the SS and SL configuration), including a reduction in some of the urinary metabolites. However, the OL configuration is only an experimental ensemble at the present time and its use by the fire service should be balanced with the potential for increased heat stress and challenges donning and doffing the gear. Additionally, short sleeves and shorts under turnout gear (SS configuration) also appeared to impact exposure, as firefighters wearing this ensemble had higher post-fire urinary concentrations of some metabolites (i.e., 1-NAP, PhGA).

## Figures and Tables

**Table 1:** Timeline for participants conducting firefighting activities in the fireground exposure simulator (FES).

Time (min)	Human Subjects	Burn Scenario
0:00	Stairs	Background–Exposure doors open
1:00		
2:00	Transition to FES & Rest	
3:00	Rest	Close Exposure Doors, Ignition
4:00	Search	
5:00		
6:00	Rest	
7:00		
8:00	Hose advance	
9:00		Suppression (~15 s)
10:00	Rest	Open front burner door
11:00		
12:00	Overhaul	Open exposure doors to vent
13:00		
14:00	Leave chamber for post-test Half of the firefighters’ unzip jacket	
15:00	Travel to data collection tent Other half of the firefighters’ unzip jacket	
16:00		
17:00		
18:00		
19:00		

**Table 2:** VOC and naphthalene metabolites and associated parent compounds.

Acronym	Analyte	Parent Compound
3HPMA	N-Acetyl-S-(3-hydroxypropyl)-L-cysteine	Acrolein
MUCA	trans, trans-Muconic acid	Benzene
PhMA	N-Acetyl-S-(phenyl)-L-cysteine	Benzene
MADA	Mandelic acid	Styrene
BzMA	N-Acetyl-S-(benzyl)-L-cysteine	Toluene or benzyl alcohol
3MHA + 4MHA	3-Methylhippuric acid + 4-Methylhippuric acid	Xylenes
2MHA	2-Methylhippuric acid	Xylenes
4HBeMA	N-Acetyl-S-(4-hydroxy-2-buten-1-yl)-L-cysteine	1,3-Butadiene
2CaEMA	N-Acetyl-S-(2-carbamoylethyl)-L-cysteine	Acrylamide
MCaMA	N-Acetyl-S-(N-methylcarbamoyl)-L-cysteine	N, N-Dimethylformamide; Methyl isocyanate
2CoEMA	N-Acetyl-S-(2-carboxyethyl)-L-cysteine	Acrolein
2CyEMA	N-Acetyl-S-(2-cyanoethyl)-L-cysteine	Acrylonitrile
2HEMA	N-Acetyl-S-(2-hydroxyethyl)-L-cysteine	Acrylonitrile; vinyl chloride; ethylene oxide
HPM2	N-Acetyl-S-(2-hydroxypropyl)-L-cysteine	Propylene oxide
3HMPMA	N-Acetyl-S-(3-hydroxypropyl-1-methyl)-L-cysteine	Crotonaldehyde
PhGA	Phenylglyoxylic acid	Ethylbenzene; styrene
1-NAP	1-hydroxynaphthalene	Naphthalene
2-NAP	2-hydroxynaphthalene	Naphthalene

**Table 3:** Benzene, toluene, styrene, and naphthalene air concentrations collected from outside and inside firefighter jackets, pants, hoods, responding to controlled fire response in the FES, stratified by PPE configuration and zip status.

Analyte (Units)	PPE	Inside/Outside	Sample Location/Type	Zip	N	N of Non-Detects	Mean	Median	Range ^A^	Workplace Protection Factors (WPFs)
Benzene (ppb)	*SS*	Outside	Active		15	0	35,878	31,177	9400–114,383	Reference
		Inside	Hood (passive)	Zip	8	0	38,822	36,538	15,865–76,923	0.85
				Unzip	7	0	41,415	26,442	19,231–120,192	1.18
			Jacket (active)	Zip	6	0	24,272	19,319	10,307–46,580	1.61
				Unzip	8	0	23,028	18,101	10,185–66,318	1.72
			Pant (passive)	Zip	10	0	37,115	32,692	16,827–67,308	0.95
				Unzip	12	1	46,314	32,452	1.68–125,000	0.96
	*SL*	Outside			14	0	54,988	56,281	16,641–98,709	Reference
		Inside	Hood (passive)	Zip	5	0	70,577	76,923	45,192–86,538	0.73
				Unzip	8	0	53,966	44,952	18,750–105,769	1.25
			Jacket (active)	Zip	6	0	40,020	48,148	14,243–53,518	1.17
				Unzip	8	0	30,900	22,897	9406–66,054	2.46
			Pant (passive)	Zip	11	0	41,587	36,058	245.2–67,308	1.56
				Unzip	11	0	45,848	30,769	16,827–100,962	1.83
	*OL*	Outside			22	0	44,732	33,744	11,810–125,333	Reference
		Inside	Hood (passive)	Zip	10	0	42,981	40,385	14,904–91,346	0.84
				Unzip	8	0	35,036	30,529	14,423–100,962	1.11
			Jacket (active)	Zip	10	0	27,329	21,358	9539–53,599	1.58
				Unzip	7	0	17,668	13,432	7225–52,042	2.51
			Pant (passive)	Zip	11	0	39,292	33,173	15,865–76,923	1.02
				Unzip	8	0	34,375	27,885	18,750–76,923	1.21
Toluene (ppb)	*SS*	Outside	Active		15	0	1016	811.3	231.8–3075	Reference
		Inside	Hood (passive)	Zip	8	0	873.4	785.9	239.5–1834	1.03
				Unzip	7	0	742.1	598.8	217.1–1984	1.35
			Jacket (active)	Zip	6	0	637.7	483.4	230.8–1316	1.68
				Unzip	8	0	549.9	416.6	243.2–1587	1.95
			Pant (passive)	Zip	10	0	755.6	711.1	239.5–1347	1.14
				Unzip	12	1	1049	654.9	40.62–3031	1.24
	*SL*	Outside			14	0	1592	1544	423.2–3347	Reference
		Inside	Hood (passive)	Zip	5	0	1594	1722	823.4–2246	0.90
				Unzip	8	0	1091	729.8	262.0–2657	2.12
			Jacket (active)	Zip	6	0	1061	1228	352.2–1495	1.26
				Unzip	8	0	766.9	487.2	207.3–1976	3.17
			Pant (passive)	Zip	11	1	874.7	1085	40.62–1572	1.42
				Unzip	11	0	1039	636.2	239.5–3481	2.43
	*OL*	Outside			22	0	1266	882.0	333.7–4347	Reference
		Inside	Hood (passive)	Zip	10	0	750	542.66	153.4–1722	1.63
				Unzip	8	0	567.9	411.7	202.1–1946	2.14
			Jacket (active)	Zip	10	0	649.9	510.2	198.0–1201	1.73
				Unzip	7	0	348.7	233.6	149.9–1029	3.78
			Pant (passive)	Zip	11	0	695.1	598.8	310.6–1272	1.47
				Unzip	8	0	556.2	430.3	243.3–1572	2.05
Styrene (ppb)	*SS*	Outside	Active		15	3	1018	758.4	33.34–3975	Reference
		Inside	Hood (passive)	Zip	8	0	433.9	390.6	91.15–989.6	1.94
				Unzip	7	0	410.3	338.5	104.2–1328	2.24
			Jacket (active)	Zip	6	4	296.4	23.12	22.63–1019	32.80
				Unzip	8	5	276.2	22.93	22.69–1331	33.07
			Pant (passive)	Zip	10	0	370.8	325.5	83.33–703.1	2.33
				Unzip	12	1	516.8	286.5	21.74–2292	2.65
	*SL*	Outside			14	1	1832	1595	32.91–4230	Reference
		Inside	Hood (passive)	Zip	5	0	828.1	859.4	442.7–1146	1.86
				Unzip	8	0	529.0	369.8	72.92–1276	4.31
			Jacket (active)	Zip	6	1	817.0	965.3	23.47–1368	1.65
				Unzip	8	4	454.6	171.8	22.32–1748	9.28
			Pant (passive)	Zip	11	1	413.0	494.8	21.74–833.3	3.22
				Unzip	11	0	482.2	252.6	75.52–1745	6.31
	*OL*	Outside			22	4	1334	830.6	33.37–5554	Reference
		Inside	Hood (passive)	Zip	10	1	224.8	183.6	21.74–572.9	4.52
				Unzip	8	1	146.9	65.10	21.74–599.0	12.76
			Jacket (active)	Zip	10	5	295.2	236.2	22.58–715.4	3.52
				Unzip	7	6	71.45	23.26	22.56–361.4	35.71
			Pant (passive)	Zip	11	1	233.0	250.0	21.74–494.8	3.32
				Unzip	8	0	192.7	170.6	65.10–546.9	4.87
Naphtha- lene (μg/m^3^)	*SS*	Outside	Active		15	0	10,607	8206	1705–44,563	Reference
		Inside	Hood (passive)	Zip	8	0	1065	787.4	397.5–2293	10.42
				Unzip	7	0	1745	886.8	412.8–6422	9.25
			Jacket (active)	Zip	6	4	479.6	132.4	129.6–1546	61.98
				Unzip	8	5	556.7	132.9	130.2–2493	61.75
			Pant (passive)	Zip	10	7	196.7	27.56	27.56–703.3	297.75
				Unzip	12	5	1942	182.0	27.56–19,877	45.09
	*SL*	Outside			14	0	16,236	16,189	2215–32,695	Reference
		Inside	Hood (passive)	Zip	5	0	3144	3058	733.9–5504	5.29
				Unzip	8	2	1784	1216	27.56–5351	13.31
			Jacket (active)	Zip	6	1	1376	1573	134.4–2010	10.29
				Unzip	8	5	893.1	132.6	127.9–4345	122.09
			Pant (passive)	Zip	11	3	556.6	412.8	27.56–1682	39.22
				Unzip	11	5	432.3	489.3	27.56–917.4	33.09
	*OL*	Outside			22	0	13,399	8344	1800–47,799	Reference
		Inside	Hood (passive)	Zip	10	8	162.7	27.56	27.56–1024	302.76
				Unzip	8	6	290.2	27.56	27.56–1361	302.76
			Jacket (active)	Zip	10	9	211.5	132.3	129.3–928.2	63.07
				Unzip	7	7	132.2	133.0	129.2–133.8	62.74
			Pant (passive)	Zip	11	11	27.56	27.56	27.56–27.56	302.76
				Unzip	8	8	27.56	27.56	27.56–27.56	302.76

^A^ Lower end of the range below the limit of detection were imputed using the β-substitution method (Ganser and Hewett, 2010), which assigned values to the non-detects based on the distribution of the uncensored data.

**Table 4:** Benzene and toluene exhaled breath concentrations collected from firefighters before and after responding to a controlled fire response in the FES, stratified by PPE configuration and zip status.

Analyte (Units)	PPE Configuration	Zip	Timing	N	N of Non-Detects	GM	Median	Range	*p*-Value (Post vs. Pre by Zip, PPE, and Analyte)	*p*-Value (Post vs. Pre by PPE and Analyte)	*p*-Value (Post vs. Pre by Analyte)
Benzene (ppbv)	SS	Zip	Pre	10	4	2.36	3.02	1.38–3.87	Reference	Reference	Reference
			Post	10	0	21.20	24.2	8.35–72.59	<0.0001	<0.0001	<0.0001
		Unzip	Pre	11	5	2.06	1.38	1.38–3.99	Reference		
			Post	12	0	26.14	31.46	5.69–117.4	<0.0001		
	SL	Zip	Pre	12	5	2.33	2.78	1.38–6.41	Reference	Reference	
			Post	12	0	31.66	26.62	6.05–111.3	<0.0001	<0.0001	
		Unzip	Pre	12	4	3.04	2.54	1.38–116.1	Reference		
			Post	12	0	22.90	15.12	6.53–105.3	0.0009		
	OL	Zip	Pre	9	4	2.13	2.66	1.38–3.63	Reference	Reference	
			Post	12	1	18.02	21.78	1.38–85.9	0.0006	<0.0001	
		Unzip	Pre	8	4	1.88	1.38	1.38–3.51	Reference		
			Post	8	0	20.24	23.59	5.93–55.65	<0.0001		
Toluene (ppbv)	SS	Zip	Pre	10	3	0.50	0.93	0.09–1.23	Reference	Reference	Reference
			Post	10	1	1.11	1.38	0.09–2.15	0.0182	0.0004	<0.001
		Unzip	Pre	11	5	0.36	0.82	0.09–2.05	Reference		
			Post	12	1	1.16	1.13	0.09–3.38	0.0082		
	SL	Zip	Pre	12	6	0.36	0.47	0.09–2.15	Reference	Reference	
			Post	12	1	1.38	1.79	0.09–3.9	0.0040	0.0110	
		Unzip	Pre	12	4	0.66	0.87	0.09–41.02	Reference		
			Post	12	3	0.76	1.23	0.09–3.49	0.6300		
	OL	Zip	Pre	9	3	0.49	0.91	0.09–1.64	Reference	Reference	
			Post	12	3	0.69	1.18	0.09–2.15	0.1572	0.0050	
		Unzip	Pre	8	6	0.17	0.09	0.09–0.93	Reference		
			Post	8	2	0.75	1.08	0.09–3.08	0.0352		

**Table 5:** Correlation between outside and inside firefighter jacket air samples (ppb) and the change in pre- and post-fire exhaled breath (ppbv) and urinary benzene metabolite concentrations (µg/g creatinine).

Outcome	Change in Pre- to Post-Fire Exhaled Breath Benzene Concentration			Pearson Correlation Coefficient	Change in Pre- to 3-h Post-Fire PhMA Urinary Concentration			Pearson Correlation Coefficient
Covariate	Estimate	SE	*p*-Value		Estimate	SE	*p*-Value	
Outside Gear Samples	0.00085	0.00012	<0.001	0.685	4.9 × 10^−6^	7.9 × 10^−7^	<0.001	0.540
SS	0.00101	0.00012	<0.001	0.910	4.9 × 10^−6^	1.7 × 10^−6^	0.010	0.605
SL	0.00121	0.00046	0.019	0.676	5.8 × 10^−6^	2.9 × 10^−6^	0.067	0.611
OL	0.00055	0.00015	0.001	0.638	4.0 × 10^−6^	1.5 × 10^−6^	0.015	0.489
Inside Jacket Samples	0.00126	0.00023	<0.001	0.672	6.7 × 10^−6^	1.4 × 10^−6^	<0.001	0.521
SS	0.00150	0.00027	<0.001	0.835	8.7 × 10^−6^	2.5 × 10^−6^	0.004	0.674
SL	0.00165	0.00091	0.127	0.690	8.8 × 10^−6^	3.4 × 10^−6^	0.023	0.565
OL	0.00087	0.00031	0.011	0.553	6.5 × 10^−6^	3.0 × 10^−6^	0.045	0.431

**Table 6:** Biomarker results (µg/g creatinine) for the firefighters conducting fire training activities in the FES.

Biomarker (Parent Chemical)	Collection Period	N (N of Non-Detects)	GM	Median	Min-Max	*p*-Value ^A^	General Population 95% Percentile (Non-Smoker/Smoker) ^B,C^	Biological Exposure Indices (BEI) ^D^
3HPMA (Acrolein)	Pre	68 (0)	223.7	209.5	79.44–1486	Reference 0.1034	835/2579	Not Available
	3 h	68 (0)	255.7	243.7	100.4–967.9			
	6 h	35 (0)	366.6	342.4	124.1–1258	<0.0001		
MUCA (Benzene)	Pre	68 (3)	72.82	49.60	8.14–1699	Reference 0.0147	Not Available	500
	3 h	68 (1)	99.62	75.65	25.94–822.0			
	6 h	35 (0)	97.90	87.09	34.16–501.8	0.0008		
PhMA (Benzene)	Pre	68 (29)	0.08	0.12	0.00–0.94	Reference <0.0001	2.62/2.98	25
	3 h	68 (8)	0.31	0.33	0.02–2.04			
	6 h	35 (3)	0.44	0.54	0.02–1.69	<0.0001		
MADA (Styrene/ Ethylbenzene)	Pre	68 (0)	109.4	115.9	40.19–506.6	Reference 0.0067	299/600	400,000
	3 h	68 (5)	130.7	136.3	19.62–422.2			
	6 h	35 (0)	153.9	144.0	85.02–305.0	<0.0001		
BzMA(Toluene or benzyl alcohol)	Pre	68 (0)	5.02	4.62	1.23–188.1	Reference 0.0124	38.9/33.1	Not Available
	3 h	68 (0)	6.28	5.91	1.74–116.1			
	6 h	35 (0)	6.60	6.05	2.33–84.27	0.1173		
3MHA + 4MHA (Xylene)	Pre	68 (0)	74.59	63.48	28.94–484.3	Reference 0.0024	872/2026	1,500,000
	3 h	68 (2)	91.97	84.74	23.48–360.2			
	6 h	35 (0)	78.29	74.28	33.43–165.4	0.1017		
2MHA (Xylene)	Pre	68 (9)	13.41	12.24	3.21–101.7	Reference 0.0011	141/354	1,500,000
	3 h	68 (9)	20.15	21.51	2.77–74.01			
	6 h	35 (8)	16.99	16.13	6.34–66.12	0.0110		
4HBeMA (1,3-Butadiene)	Pre	68 (0)	4.65	4.64	1.47–11.10	Reference 0.0044	14.9/87.9	Not Available
	3 h	68 (0)	5.68	5.71	2.71–16.18			
	6 h	35 (1)	4.85	4.61	2.72–11.01	0.3105		
2CaEMA (Acrylamide)	Pre	68 (0)	60.04	64.96	10.09–207.2	Reference	141/326	
	3 h	68 (0)	63.75	60.48	16.76–233.5	0.3497		
	6 h	35 (0)	52.92	54.07	27.12–109.6	0.8786		
MCaMA- (Methyl isocyanate)	Pre	68 (0)	94.07	97.91	13.19–312.3	Reference	390/1248	
	3 h	68 (1)	92.05	90.33	9.32–351.8	0.7480		
	6 h	35 (1)	85.70	107.8	10.62–417.6	0.0177		
2CoEMA (Acrolein)	Pre	68 (0)	78.64	80.16	20.54–378.2	Reference	212/491	
	3 h	68 (0)	86.49	81.82	30.39–375.0	0.1461		
	6 h	35 (0)	93.61	90.19	42.54–289.2	0.1194		
2CyEMA (Acrylonitrile)	Pre	68 (6)	3.21	3.05	0.50–61.51	Reference	8.69/416	
	3 h	68 (0)	23.65	23.66	4.17–134.9	<0.0001		
	6 h	35 (0)	21.13	19.24	7.43–68.13	<0.0001		
2HEMA (Acrolynitrile, vinyl chloride, ethylene oxide)	68 (28) 68 (18) 35 (7)	68 (28)	0.38	0.52	0.03–4.22	Reference	3.48/9.95	
		68 (18)	0.57	0.65	0.03–4.11	0.1198		
		35 (7)	1.30	1.32	0.11–5.05	<0.0001		
HPM2 (Propylene oxide)	Pre	68 (2)	26.36	25.99	5.63–92.63	Reference	159/152	
	3 h	68 (6)	25.49	26.13	6.87–78.38	0.4000		
	6 h	35 (1)	27.73	25.35	11.94–70.63	0.6611		
3HMPMA (Crotonaldehyde)	Pre 3 h 6 h	68 (0)	171.0	143.0	85.62–551.8	Reference	1450/5327	
		68 (0)	266.1	245.0	84.07–1530	<0.0001		
		35 (0)	349.3	380.8	171.1–661.7	<0.0001		
PhGA (Styrene, ethylbenzene)	Pre 3 h 6 h	68 (0)	173.6	172.7	51.47–452.4	Reference	377/764	
		68 (0)	197.9	194.0	79.02–423.5	0.0228		
		35 (0)	199.7	208.5	29.90–526.6	0.0271		
1-NAP (Naphthalene)	Pre 3 h 6 h	68 (0)	0.88	0.91	0.19–6.44	Reference	18.3/41.4	
		68 (0)	3.46	3.26	0.50–39.96	<0.0001		
		35 (0)	2.77	2.84	0.65–7.17	<0.0001		
2-NAP (Naphthalene)	Pre 3 h 6 h	68 (0)	6.63	7.02	0.57–33.65	Reference	18.3/34.1	
		68 (0)	15.49	19.82	2.59–77.72	<0.0001		
		35 (0)	10.65	11.33	2.84–41.53	0.0008		

^A^ Test of significant geometric mean (GM) difference between pre- and 3-h or 6-h post-fire concentrations. ^B^ National Health and Nutrition Examination Survey (NHANES) (2018). 2013–2014 data documentation, codebook, and frequencies. Volatile Organic Compounds & Metabolites—Urine (UVOC_H). Available at https://wwwn.cdc.gov/Nchs/Nhanes/2013-2014/UVOC_H.htm (accessed on 10 October 2022). ^C^ National Health and Nutrition Examination Survey (NHANES) (2018). 2015–2016 data documentation, codebook, and frequencies. Polycyclic aromatic hydrocarbons (PAHs)—Urine (PAHS_I). Available at https://wwwn.cdc.gov/Nchs/Nhanes/2015-2016/PAHS_I.htm (accessed on 15 November 2022). ^D^ American Conference of Governmental Industrial Hygienists (ACGIH) (2021). “2021 TLVs and BEIs with 9th Edition Documentation”. Cincinnati, OH, USA: ACGIH.

## Data Availability

All available data have been reported in this manuscript.
